# Anti-aging effect of low molecular weight recombinant humanized collagen on photo-aging by activating adherence junction signaling pathways

**DOI:** 10.1371/journal.pone.0329460

**Published:** 2025-08-29

**Authors:** Kan Tao, Huilin Zhu, Jing Wei

**Affiliations:** 1 Research and Development Department, Shanghai Chicmax Cosmetic Co., Ltd., Putuo District, Shanghai, China; 2 Nanjing Vazyme biological pharmaceutical Co., Ltd., Nanjing, China; 3 Nanjing Dynova Biotech Co., Ltd., Nanjing, China; University of Waterloo, CANADA

## Abstract

Skin aging is characterized by a loss of collagen. Collagen stimulates the secretion of extracellular matrix (ECM) components by skin fibroblasts, contributing to anti-wrinkle and skin-firming effects in cosmetic applications. However, the skin barrier poses a significant challenge to collagen absorption, hindering its dermal functionality. Rapid advancements in synthetic biology have enabled the development of recombinant human collagen (RHC) with controllable sequence and molecular weight, enhancing its potential cosmetic applications. Nonetheless, research on the ability of RHC to penetrate the skin and exert anti-aging effects remains limited, and its underlying mechanisms are largely unexplored. To address this gap, we selected low molecular weight recombinant human collagen peptide (LRHC) and evaluated its skin permeability and anti-aging mechanisms. Findings indicated that LRHC significantly promoted fibroblast proliferation and enhanced the transcription of collagen types I and type III. Furthermore, in photoaged nude mouse models, LRHC upregulated the expression of key basement membrane components, including collagen type IV (COL4), collagen type VII (COL7), collagen type XVII (COL17), integrin β4 (ITGB4), and laminin-332 (LN332, formerly LN5), resulting in increased collagen fiber density. Notably, LRHC demonstrated a dermal permeability rate of 74.7 ± 14.2% after 8 hours. Transcriptome sequencing revealed that LRHC may maintain cytoskeletal structure through activation of adherens junction signaling pathways and promote extracellular matrix (ECM) production through activation of transforming growth factor-beta (TGF-β) signaling pathways, thereby achieving anti-aging efficacy.. These findings confirm that LRHC can penetrate the dermis and exert anti-aging effects on skin, potentially through mechanisms mediated by adherens junction signaling pathways. As a functional active ingredient with anti-aging properties, LRHC holds significant potential for cosmetic applications.

## Introduction

Photoaging, the premature aging of human skin, is induced by continuous exposure to ultraviolet (UV) radiation and is characterized by wrinkles and loss of elasticity [1]. Expression of matrix metalloproteases (MMPs) in human skin is increased by UV radiation. MMPs degrade extracellular matrix (ECM) proteins, including collagen, fibronectin, elastin, and proteoglycans [2]. Reduction of dermal ECM proteins in the dermis is the primary mechanism underlying dermal atrophy, which results in reduced skin elasticity and increased wrinkle formation [3]. Therefore, effective strategies to promote collagen regeneration and mitigate skin aging are required.

Collagen fibers, constituting the primary structural component of the dermis, represent approximately 80% of the dermal connective tissue and 72% of the skin’s dry matter [4]. The effect of oral collagen supplementation and injectable collagen administration on skin aging improvement have been extensively studied, and these approaches have become established representative anti-aging strategies [5–8]. Oral administration of hydrolyzed collagen has been demonstrated in human studies to improve skin hydration and elasticity [9]. Following intradermal injection of tilapia skin collagen into photoaged Kunming mice, skin aging was effectively alleviated [10]. Limitations associated with orally delivered bioactive peptides include instability within the gastrointestinal tract, poor intestinal mucosal permeability, and rapid systemic metabolism [11]. Injectable administration is also associated with well-known drawbacks, including procedural pain and poor patient acceptability, as well as concerns related to immunogenicity and potential viral contamination cannot be ignored [5,12,13].

The daily application of cosmetics represents a widely adopted method for decelerating the skin aging process. Collagen is utilized as a cosmetic active ingredient due to its excellent moisturizing and anti-aging properties [14,15]. However, the development of collagen-based cosmetics has progressed more slowly than that of injectable fillers and oral supplements. This limitation is primarily attributed to the high molecular weight (~300 kDa) of native collagen, which results in low skin permeability and consequently limited efficacy [16]. Hydrolysis offers a solution for reducing collagen molecular weight, with enzymatic hydrolysis being the most extensively studied method [17–20]. Nonetheless, hydrolyzed collagen typically constitutes a polydisperse mixture, which compromises batch-to-batch consistency and hampers mechanistic investigations. Recombinant collagen production utilizing synthetic biology has emerged as an efficient and sustainable alternative, with significant advancements achieved in recent years [21–25]. However, the ability of recombinant collagen to penetrate the dermis and promote extracellular matrix (ECM) regeneration remains poorly characterized, and its mechanism of action remains unclear. We chose RHCare Micro, a low molecular weight recombinant human collagen peptide (LRHC) exhibiting a homogeneous molecular weight distribution. This material is produced using synthetic biology followed by specific enzymatic hydrolysis technology, we investigated its permeability, anti-aging properties, along with the underlying mechanism of action.

## Materials and methods

### Preparation of LRHC

The DNA fragment encoding recombinant human type III collagen (hlcollagen) was cloned into the prokaryotic expression vector pET-3c (Invitrogen), generating the recombinant plasmid pET3c-hlcollagen. This plasmid was then transformed into E.coli BL21(DE3) competent cells (Invitrogen). Protein expression was induced by adding 1 mM IPTG to the culture, which was further incubated at 37°C for 4 hours. Bacterial cells were harvested by centrifugation, resuspended in lysis buffer, and disrupted using a high-pressure homogenizer. The recombinant protein was purified using a two-step chromatography protocol: Ni-affinity chromatography (Ni Sepharose 6 Fast Flow) to capture the His-tagged protein, followed by size-exclusion chromatography (Sephadex G-25) for buffer exchange and final purification. The purified recombinant human type III collagen was collected and concentrated.

The aqueous solution of type III recombinant humanized collagen (10 mg/mL) was denatured *via* high-temperature treatment in a constant-temperature water bath at 80–100°C for 10–60 minutes to loosen the protein structure, yielding Digestion Product I. The aqueous solution of type III recombinant humanized collagen (10 mg/mL) was subjected to high-temperature denaturation at 80–100°C in a constant-temperature water bath for 10–60 minutes to loosen the protein structure, digestion product I was obtained. ‌Digestion product I was cooled to room temperature, its pH was adjusted to 4–10, and protease was added at 0.005%−0.05% by weight (relative to the substrate). It was then subjected to enzymatic digestion with stirring at 37°C in a constant-temperature incubator for 2–40 hours, yielding Product II. ‌Product II was inactivated at 70–100°C in a constant-temperature water bath for 2 hours. It was then filtered through a 0.22 μm membrane to obtain low molecular weight recombinant humanized collagen (LRHC).

The molecular weight of LRHC was characterized via SDS-PAGE, and its purity was analyzed by high-performance liquid chromatography (HPLC).

### HPLC determination

The control product was precisely weighed and dissolved in ultrapure water to prepare a stock solution of 10 mg/mL. Subsequently, both the LRHC sample and the control product were precisely diluted to 1 mg/mL using the mobile phase. The LRHC sample solution and control solution were analyzed under the following conditions, respectively. The chromatography was performed on BioCore SEC-150 (7.8 × 300 mm, 5 um) column with 150 mM phosphate buffer (PB, pH 7.0) as the mobile phase. The column temperature was set at 30°C and the flow rate was set at 1 mL/min. The detection wavelength was 204 nm, and the injection volume was 5 μL. Chromatograms were recorded using a suitable data acquisition system.

### SDS-polyacrylamide gel electrophoresis (SDS-PAGE)

Tricine-SDS-PAGE was conducted using a Tricine-SDS-PAGE Gel Preparation Kit (Sango Biotech, China). Ssamples were diluted to 2 μg or 4 μg per lane for loading, heated at 95°C for 10 min for denaturation. Electrophoresis was carried out at 150 V for 80 min. Gels were stained with Coomassie Brilliant Blue at room temperature for 10 minutes and destained until clear protein bands were visible.

### Cell lines and culture procedure

The human fibroblasts (FBs) were obtained from Guangzhou Biotechnology (PC2031) and cultured in Dulbecco’s Modified Eagle Medium (DMEM, Gibco, C11885500BT) supplemented with 10% fetal bovine serum (Ausgenex, FBS500-S) and 1% penicillin/streptomycin. The NIH/3T3 cell line (American type culture collection, ATCC, CRL-1658) and the HaCaT cell line (BeNa Culture Collection, BNCC339817) were cultured in DMEM (Gibco, C11995500BT) supplemented with 10% fetal bovine serum and 1% penicillin/streptomycin. All cells were maintained in a humidified atmosphere containing 5% CO_2_ at 37°C.

### Cell counting kit-8 (CCK8) assay

FBs were seeded in 96-well plates at a density of 1 × 10⁴ cells/well and cultured overnight to allow attachment. Then treated with LRHC or bovine collagen type Ⅰ (positive control) (Solarbio, C8061) in serum-free medium for 24 h, under the indicated conditions (37°C, 5% CO₂). Cell proliferation was measured using a CCK8 kit (Vazyme, A311-01), according to the manufacturer’s protocol. The absorbance of the live cells was measured at 450 nm using a microplate spectrophotometer (Tecan, Spark).

### Wound scratch assay

HaCaT cells were seeded into 12-well plates at a density of 3 × 10^5^ cells/well and incubated at 37°C in 5% CO2. After incubation for 24 h, a scratch wound was created using a sterilized 200-µL pipette tip. The cells were washed with phosphate-buffered saline (PBS), and serum-free DMEM containing LRHC or bovine collagen type I was added to each well. Images of the scratched areas from three independent experiments were acquired at 0 and 24 h using a biological photomicroscope (Olympus, IX73). The area of cell migration was quantified using Image-J software, by calculating the ratio of the remaining scratch area at 24 h to the original scratch area at 0 h. At least three microscopic fields were analyzed for each condition.

### Cell adhesion assay

LRHC and bovine collagen type I (positive control) were used to coat 96-well plates at 4°C overnight, followed by rinsing with 1% heat-denatured BSA solution to block non-specific binding sites. NIH/3T3 cells were seeded into the coated plates at a density of 1 x 10^5^ cells/well in serum-free culture medium and incubated at 37°C in 5% CO_2_ for 1 h. The culture medium was removed from all wells except three designated for “Total Cell” count. Cells were rinsed with PBS 3 times, and then 100 µL serum-free culture medium was added per well after rinsing. Cell adhesion was assessed using the CCK8 kit to determine the number of cells adherent to the coated plates. The adhesion rate was calculated as the ratio of adherent cells to total cells seeded.

### Ultraviolet A (UVA) irradiation on FBs

FBs were seeded at a density of 4 × 10^5^ cells/well into 6-well plates and incubated in growth medium until 70–80% confluence. Following washing with phosphate-buffered saline (PBS), cells were exposed to ultraviolet A radiation at a dose of 5 J/cm^2^ using a UV crosslinker (Scientz, 03-II) with a spectral peak at 365 nm. After UVA irradiation, PBS was replaced with the medium containing HC, and incubated at 37°C in 5% CO_2_ for 24 h.

### UVA irradiation on BALB/c-nude mice

This study was carried out in strict accordance with the recommendations in the Guide for the Care and Use of Laboratory Animals of the National Institutes of Health. All animal experiments, animal care and animal model protocols were approved by the Institutional Animal Care and Use Committee (IACUC) of Nanjing Vazyme Biotech Co., Ltd. under protocol number LAE-2024005. We obtained 8-week-old female Balb/c nude mice from Yangzhou University. The experiment was conducted on the back of each mouse. The animal experiment was conducted over 2 weeks, and the health and behavior of the animals were monitored daily (excluding weekends). Only mild pain or distress was anticipated during irradiation. Upon the conclusion of the two-week experimental period, all mice were humanely euthanized *via* CO_2_ inhalation after halothane anesthesia.

The mice were irradiated with UVA at 10 J/cm^2^, 3 times a week for 2 weeks (a total dose of 60 J/cm^2^). LRHC (dissolved in Vaseline) was topically applied to a 1 cm^2^ area of the shaved dorsal skin once a day during the 2 weeks of UVA exposure. All mice were euthanized by CO_2_ inhalation after halothane anesthesia after 24 hours after the last irradiation. The dorsal skin flaps were excised and a portion was stored in 4% paraformaldehyde for RT-qPCR and Masson assay.

### RNA extraction and qRT-PCR

Total RNA was extracted using Trizol Reagent (Vazyme, RH103-C1) following the manufacturer’s protocol. HiScript II One Step qRT-PCR Probe Kit (Vazyme, Q222-01) was used for the reverse transcription and qPCR according to the manufacturer’s instructions. Set up the PCR cycle conditions by referring to the instructions in the HiScript II One Step qRT-PCR SYBR Green Kit. To analyze the relative expression level of the mRNA, the 2 (−∆∆Ct) method was used, with normalization to GAPDH. The specific primer sequences used in this study are listed in Table 1.

**Table 1:** Primer sequences.

Species	Gene	Forward Primer (5’ to 3’)	Reverse Primer (5’ to 3’)
Human	COL1	GAGGGCCAAGACGAAGACATC	CAGATCACGTCATCGCACAAC
	COL3	GGAGCTGGCTACTTCTCGC	GGGAACATCCTCCTTCAACAG
	ELN	GCAGGAGTTAAGCCCAAGG	TGTAGGGCAGTCCATAGCCA
	TGF-β	CCCCGGAGGTGATTTCCATC	GGGCGGCATGTCTATTTTGTAAA
	Smad2	CCGACACACCGAGATCCTAAC	GAGGTGGCGTTTCTGGAATATAA
	GADPH	ACTCCCACTCTTCCACCTTC	TCTTGCTCAGTGTCCTTGC
Mouse	COL4	CTGGCACAAAAGGGACGAG	ACGTGGCCGAGAATTTCACC
	COL7	ACCACGTTTCTGACCGTGTC	AGCTGTGTCCACTAAATCTTGG
	COL17	ATGACTTCAGAGGGATCATCAACAATCACT	AGTGAAGAAGGTTTCTGAGTCAGTCAG
	ITGB4	AAGTCCAACTCAGCAACCCC	CTCCTGTCCGTTTCATCGAG
	LN5	ACCTGGCTTTGACGGTCCT	GTTGAAGCCAAAGCGTACAGCG
	MMP9	GCAGAGGCATACTTGTACCG	TGATGTTATGATGGTCCCACTTG
	GADPH	ACTCCCACTCTTCCACCTTC	TCTTGCTCAGTGTCCTTGC

### Paraffin sectioning

Fix the dissected tissue in 4% paraformaldehyde on ice or at 4°C for 24 h. Wash the tissue twice in PBS for 5 minutes each. Dehydrate the tissue in a graded ethanol series: 50% ethanol, 70% ethanol, 80% ethanol, 95% ethanol, and 100% ethanol twice, followed by two changes of xylene. Tissues were then infiltrated with paraffin by incubation in a 1:1 mixture of paraffin and xylene at 56–60°C under vacuum for 30 min, followed by two changes of pure paraffin under the same conditions. Embed the tissue in molten paraffin in a metal mold and allow it to cool at room temperature. Blocks can be stored at 4°C for several months. Cut the tissue into 5–6 µm sections using a microtome, float them in a water bath containing 10% ethanol (EtOH), transfer to silane-coated slides, and dry overnight at 42°C. Sections can be stored at 4°C in a desiccator for several months. Prior to staining, sections were dewaxed by two 10-minute incubations in xylene and rehydrated through a descending ethanol series (100%, 95%, 90%, 80%, 70%, 50%, 30%) followed by two 5-minute washes in phosphate-buffered saline (PBS). For immunohistochemistry, sections were blocked for 30 minutes as usual.

### Masson’s trichrome staining

Deparaffinized and rehydrated sections were treated in preheated Bouin’s Solution at 56°C for 15 minutes or at room temperature overnight. Sections were cooled in room temperature water (18–26°C) and then washed in running tap water until the yellow color was removed from the sections. Stain the sections in Working Weigert’s Iron Hematoxylin Solution for 5 minutes. Wash in running tap water for 5 minutes. Rinse in deionized water. Stain in Biebrich Scarlet-Acid Fucshin for 5 minutes. Rinse in deionized water. Transfer slides to Working Phosphotungstic/Phosphomolybdic Acid Solution for 5 minutes. Then transfer the slides to Aniline Blue Solution for 5 minutes. Place the slides in 1% Acetic Acid for 2 minutes. Rinse slides briefly in deionized water, dehydrate through a graded ethanol series, clear in xylene, and mount with a suitable mounting medium. Images were acquired using an inverted fluorescent microscope, and collagen fiber density was quantified using ImageJ software.

### Preparation of FITC-labeling LRHC(FITC-LRHC)

LRHC and bovine collagen were dissolved in Na_2_CO_3_/NaHCO_3_ buffer (pH 9.0) to a concentration of 10 mg/mL. FITC (Aladdin, F104848) was dissolved in DMSO to prepare a stock solution with a concentration of 1 mg/mL. Immediately before use, the stock solution was diluted to 10 mM with DMSO. The LRHC and bovine collagen solution was mixed with the diluted FITC solution at a molar ratio of 1:1 (LRHC and bovine collagen to FITC). The reaction mixture was incubated in the dark at room temperature for 1 hour. To quench the reaction, NH4Cl was added to a final concentration of 50 mM and the mixture was incubated for an additional 1 hour. Unbound FITC was removed using a Desalting Column (Thermo Scientific, 89882) according to the manufacturer’s instructions. The purified FITC-LRHC and FITC-bovine collagen conjugate was stored in PBS buffer at 4°C protected from light.

### Permeation study

An *in vitro* skin permeation test was conducted using a Franz-type diffusion cell (effective diffusional area: 1.00 cm^2^, receptor volume: 10.00 mL). Skin from Bama miniature pigs was obtained from Pizhou Dongfang Breeding Co., Ltd. and stored at −20°C. For the *in vitro* experiments, the porcine skin was thawed at room temperature and placed on the Franz receptor cell, stratum corneum side up. The receptor cell (dermis side) was filled with 0.9% NaCl and maintained at 37°C with a magnetic stirrer bar. This setup ensured that the temperature at the stratum corneum surface remained at 37°C. A volume of 200 μL containing 2 mg of LRHC and bovine collagen was applied to the stratum corneum side. At different time points after application, 1.2 mL of the receptor solution was collected from the sampling port of the Franz cell, and an equal volume of PBS (pH 7.4) at 37°C was added back to the receptor cell. Fluorescence imaging was performed using an inverted fluorescent microscope, and the cumulative amount of LRHC and bovine collagen penetrating the skin was quantified using UV-VIS spectroscopy.

### RNA-seq

FBs were seeded at 4 × 10^5^ cells/well into 6-well plates and then treated with or without LRHC in serum-free medium for 24 h under the indicated conditions. Subsequently, total RNA was isolated. The concentration of the isolated RNA was determined using the Equalbit RNA BR Assay Kit (Vazyme, EQ212). RNA fragments (500 ng) from each group were purified using oligo(dT)-attached magnetic beads and then reverse-transcribed to cDNA using VAHTS Universal V10 RNA-seq Library Prep Kit for Illumina (Vazyme, NR606), according to the manufacturer’s protocol. The resulting cDNA libraries were constructed by ligating adapters and amplifying *via* PCR.The concentrated library was assessed for quantity and quality using the Equalbit 1x dsDNA HS Assay Kit (Vazyme, EQ121) and the Agilent DNA1000 Kit (Agilent, 5067−1504), following the manufacturer’s instructions. Multiple libraries were combined in equal molar ratios and subsequently sequenced on an Illumina platform (MGI2000). Adapter and low-quality sequences were removed during quality control. The quality-controlled reads were then aligned to the Homo sapiens reference genome (GRCh38, obtained from the UCSC genome browser, http://genome.ucsc.edu, accessed on 31 March 2021) using HISAT v2.1.0. Transcript-level expression abundance was quantified as read counts using StringTie-1.3.1c. Differential gene expression analysis was performed using DESeq2, with genes exhibiting an adjusted p-value < 0.05 considered differentially expressed (DEGs). Gene Ontology (GO) and KEGG pathway enrichment analyses for significant DEGs were conducted using the R package clusterProfiler.

### Statistical analysis

Statistical analyses were performed using Student’s two-tailed t-test and one-way ANOVA with Prism 7 software (GraphPad, San Diego, CA, USA). The p-values are shown in the figures. The differences were considered statistically significant at *p < 0.05, **p < 0.01, and ***p < 0.001. All representative data were obtained from triplicate experiments and are presented as mean ± standard deviation.

## Results

### Preparation and characterization of LRHC

**Fig 1:**
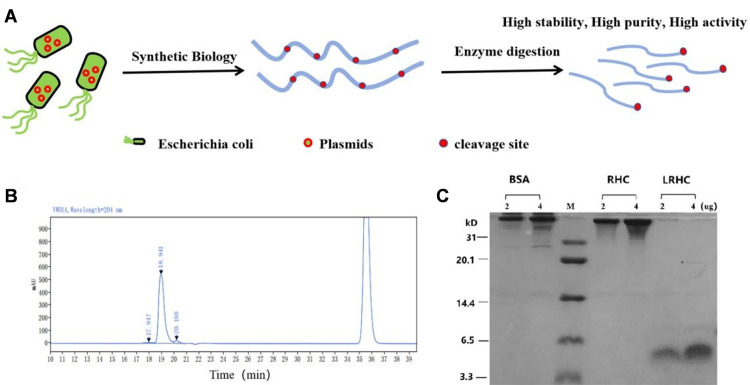
(A) Schematic diagram for preparing LRHC. (B) The HPLC chromatogram of LRHC. (C) The SDS polyacrylamide gel electrophoresis of LRHC.

In this study, LRHC with high stability, high purity, and high activity was obtained by specific enzymatic hydrolysis of type III recombinant humanized collagen (Fig 1A). The results of HPLC are shown in Fig 1B, LRHC displayed only one single main peak, with a purity of 95.7%. SDS-PAGE results showed that LRHC with low molecular weight and single band was obtained from type III recombinant humanized collagen by enzyme digestion. Meanwhile, LRHC showed only one single band in SDS-PAGE, indicating that LRHC obtained by enzymatic hydrolysis is a single component with low molecular weight (Fig 1C).

### LRHC promotes cell proliferation, migration, and adhesion

**Fig 2:**
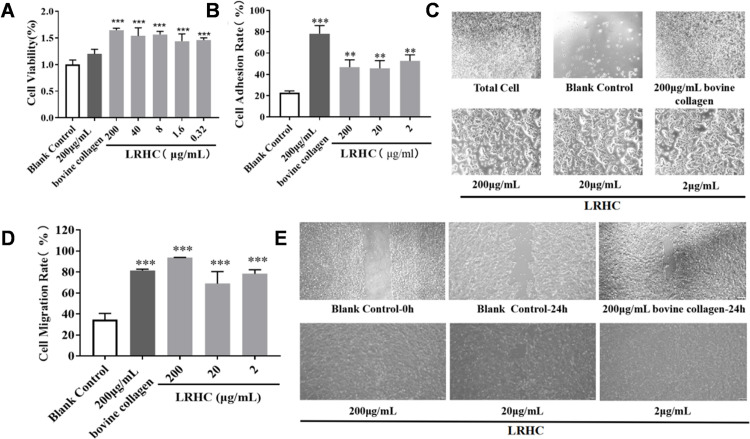
Biological activity of LRHC in promoting cell proliferation, migration, and adhesion. **(A)** Cell proliferation by CCK8 assay; **(B, C)** Adhesion activity measured by cell adhesion assay; **(D, E)** Cell migration rate measured by wound scratch assay. Statistical analyses were performed using two-way ANOVA with Tukey multiple comparisons test (A, B, and **D)**. Data represent mean ± SEM (n = 3). *p < 0.05, **p < 0.01, and ***p < 0.001 *vs.* the Blank Control.

Collagen provides mechanical support in tissues and plays an important role in controlling cell proliferation, cell adhesion, cell migration, and tissue repair [26,27]. LRHC demonstrated concentration-dependent proliferative effects on cells. As shown in Fig 2A, cell viability was increased to 1.66 ± 0.04-fold of blank control by treatment with 200 μg/mL LRHC., and the cell viability was increased to 1.46 ± 0.04-fold of blank control with 0.32 μg/mL LRHC. We evaluated the adhesive properties of LRHC using mouse NIH/3T3 fibroblasts. As illustrated in Fig. 2B, 2C, LRHC exhibited no significant concentration-dependent effects on cell adhesion across the 20–200 μg/mL range. The positive control (200 μg/mL bovine type I collagen) demonstrated a cell adhesion rate of 78.22 ± 7.59%. Notably, 2 μg/mL LRHC-coated surfaces promoted NIH/3T3 cell adhesion to 52.55 ± 5.77%, representing a statistically significant enhancement compared to blank control (22.97 ± 1.46%). The cell scratch assay (Fig 2D, 2E) demonstrated that LRHC significantly enhanced HaCaT cell migration. Treatment with 200 μg/mL LRHC increased the migration rate to 93.76 ± 0.11%, outperforming both the positive control (200 μg/mL bovine type I collagen: 81.4 ± 1.31%) and negative control (34.58 ± 5.98%).

### LRHC promotes ECM expression in UVA-exposed FBs

**Fig 3:**
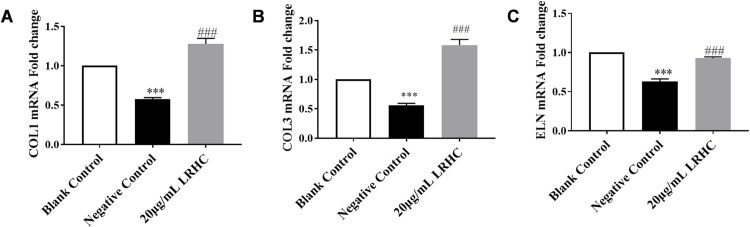
The influence of LRHC in ECM proteins in FBs. The levels of **(A)** COL1 mRNA, **(B)** COL3 mRNA, and **(C)** ELN mRNA are measured by RT-qPCR assay. Statistical analyses were performed using a two-way ANOVA with Tukey multiple comparisons test. Data are presented as mean ± SEM (n = 3). *p < 0.05, **p < 0.01 *vs.* the Blank Control, ##p < 0.01, and ###p < 0.001 *vs.* the Negative Control.

To further investigate ECM transcriptional regulation, we analyzed gene expression in fibroblasts (FBs) using RT-qPCR. As shown in Fig 3, UVA irradiation significantly suppressed the transcription of key ECM components, including COL1 (0.57 ± 0.02), COL3 (0.56 ± 0.03), and ELN (0.63 ± 0.06), compared to untreated control. Strikingly, LRHC treatment reversed this inhibition, markedly upregulating COL1 (1.28 ± 0.07; 2.2-fold increase), COL3 (1.58 ± 0.10; 2.8-fold increase), and ELN (0.93 ± 0.03; 1.5-fold increase) relative to UVA-exposed cells.

### LRHC improves the skin aging induced by UVA in Nude Mice

**Fig 4:**
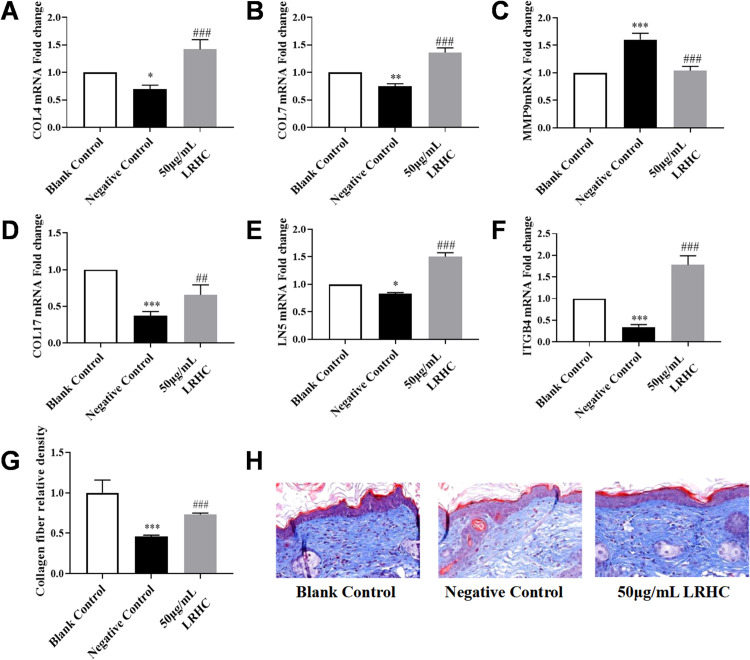
The influence of LRHC in basement membrane relative proteins and collagen fiber density on balb/c-nude mice. **(A-F)** The influence of LRHC in basement membrane relative proteins measured by RT-qPCR. **(G-H)** The influence of LRHC on dermal fiber density by Masson assay. Statistical analyses were performed using a two-way ANOVA with Tukey multiple comparisons test **(A-G)**. Data represent mean ± SEM(n = 3). *p < 0.05, **p < 0.01 *vs.* the Blank Control, ##p < 0.01, and ###p < 0.001 *vs.* the Negative Control.

The skin basement membrane is located at the true epidermis junction. The basement membrane separates and connects the dermal and epidermis, exchanging signal, nutrients, oxygen, and waste products between these two layers. The basement membrane is considered to be closely associated with the aging process. Its primary composition includes COL4, COL7, COL17, LN5, and ITGB4, while MMP9 acts on the enzymatic degradation of COL4 [28–30]. As demonstrated in Figs 4a–4f, a significant reduction in the expression levels of COL4, COL7, COL17, LN5, and ITGB4 was observed in the skin of Balb/c nude mice following UVA irradiation, while MMP9 mRNA expression was found to be upregulated. After local administration of LRHC, marked improvements in the mRNA expression of these critical basement membrane components (COL4, COL7, COL17, LN5, and ITGB4) were achieved compared to the negative control group. Concurrently, MMP9 expression was restored to levels equivalent to those detected in the blank control group.

Collagen fibers, serving as critical structural components of skin tissue, are significantly reduced with aging, leading to decreased skin elasticity and increased laxity. Through Masson, s trichrome staining combined with ImageJ software analysis (Figs 4G–4H), the stained area was quantified for each tissue sample. following UVA exposure, a marked decline in relative collagen fiber density was observed in the dermal tissue of BALB/c nude mice, reaching only 0.46 ± 0.011 compared to the blank control group. However, after treatment with 50 μg/mL LRHC, a significant restoration in collagen fiber density was achieved, returning to 0.73 ± 0.02 of the control level.

### LRHC perpetuates skin barrier integrity through molecular stabilization

**Fig 5:**
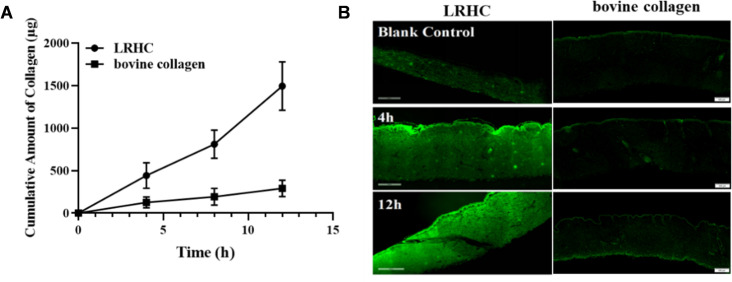
The skin permeability of LRHC and bovine collagen. **(A)** The Permeation LRHC and bovine collagen was using Franz-type diffusion cell, and UV-VIS was used to quantify the cumulative amount of LRHC in the receptor cells. Results are expressed as the mean ± SEM (n = 3). **(B)** The retention of FITC-LRHC and FITC-bovine collagen in the skin was observed by fluorescence microscopy through paraffin sections, scale bar = 500 μm.

Fluorescence microscopy imaging revealed intense fluorescence in the epidermal layer but minimal signal in the dermis. These observations indicate that LRHC primarily accumulates within the epidermal layer, with only trace amounts penetrating into the dermis. By 12 h, the strong fluorescent signal in the dermis demonstrated substantial penetration of LRHC into this layer. However, bovine collagen (higher molecular weight) exhibited persistently weak dermal fluorescence even after 12 hours, suggesting its limited penetration capability. As shown in Fig 5A–5B, at 4 h, cumulative amounts of LRHC and bovine collagen in the receptor compartment reached 442.31 ± 150.71 μg (22.12 ± 7.54% of initial load) and 125.12 ± 36.79 μg (6.15 ± 1.84%), respectively; At 8 h: the cumulative amounts reached 810.63 ± 165.16 μg (40.53 ± 8.26%) and 193.29 ± 57.17 μg (9.66 ± 2.86%); At 12 h, 1494.5 ± 284.44 μg (74.73 ± 14.22%) and 290.92 ± 55.91 μg (14.55 ± 2.79%). Collectively, the penetration efficacy of LRHC was significantly superior to bovine collagen (*p* < 0.01).

### RNA-seq analysis revealed that LRHC stabilizes cytoskeletal architecture *via* adherens junction signaling pathways

**Fig 6:**
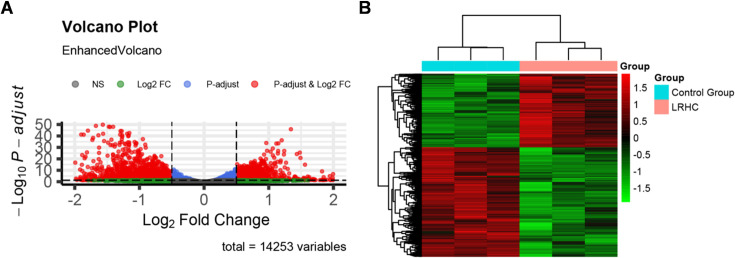
(A) Volcano plot. The horizontal coordinate is the Fold change of gene expression between the two groups, which is generally expressed by log2FC. The vertical coordinate is expressed as a -log10 (*P*) value, indicating the significance of differential gene expression. (B) Clustering heat map. The horizontal coordinate of the heat map is the sample of each group, and the vertical coordinate is genes. The redder the color in the grid, the higher the expression of the gene in the sample, and the bluer the color, the lower the gene expression.

To characterize the biological activity of LRHC, we performed transcriptomic profiling of human fibroblasts (FBs) treated with or without LRHC. Differential gene expression analysis identified 3332 significantly differentially expressed genes (DEGs), including 1339 upregulated and 1993 downregulated DEGs in LRHC-treated cells versus controls (Fig 6A, 6B). The top 10 upregulated and downregulated DEGs (ranked by *p-value*) are listed in Table 2.

**Table 2:** Representative differentially expressed genes after LRHC treatment.

Upregulated gene name	Log2FC	padj	Downregulated gene name	Log2FC	padj
*AC159540.1*	2.411704231	0.002436124	*RN7SL2*	−6.414177165	6.55E-08
*RP11-1102P16.1*	2.407113487	0.035822832	*DDT*	−4.143906436	2.18E-05
*NUDT13*	2.33857787	0.005831656	*PXN-AS1*	−3.950382355	0.000176958
*ZNF620*	2.252038064	0.002840013	*SH3RF3-AS1*	−3.168876025	0.000169501
*C1QTNF3*	2.146173513	0.020978428	*PRR13P5*	−2.904262282	0.004454585
*ZNF730*	2.075454334	0.020625476	*TMEM160*	−2.804011772	0.000433861
*TMEM155*	2.019726954	0.033735805	*AC131263.1*	−2.797854825	0.002432552
*RP1-68D18.4*	2.002099921	0.013568987	*NUDT1*	−2.79277689	9.54E-08
*CENPE*	1.99488182	2.10115E-07	*CLDN4*	−2.715105339	0.005513625
*ZNF410*	1.991417438	0.040807477	*FOXN3-AS1*	−2.711838692	0.001076075

**Fig 7:**
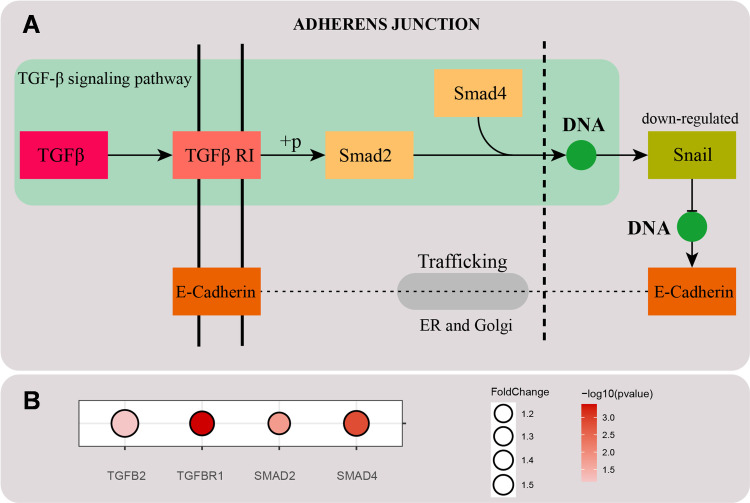
(A) The schematic diagram of adherence junction signaling pathway. Painted by Adobe Illustrator 2023. (B) Bubble map of genes in TGF-β signaling pathway. Fold Change indicates the gene expression multiple relative to the control group, *p-value* indicates the significance of genes.

KEGG enrichment analysis of upregulated DEGs revealed adherens junction as a significantly enriched pathway (*p* < 0.001) associated with extracellular matrix regulation. Within this pathway, the TGF-β signaling branch showed upregulation of core components (Fig 7A–7B), indicating pathway activation. Concomitant downregulation of SNAI1 (*p* < 0.01) attenuated E-cadherin degradation, facilitating its trafficking *via* the endoplasmic reticulum and Golgi apparatus to the plasma membrane, thereby enhancing intercellular adhesion.

**Fig 8:**
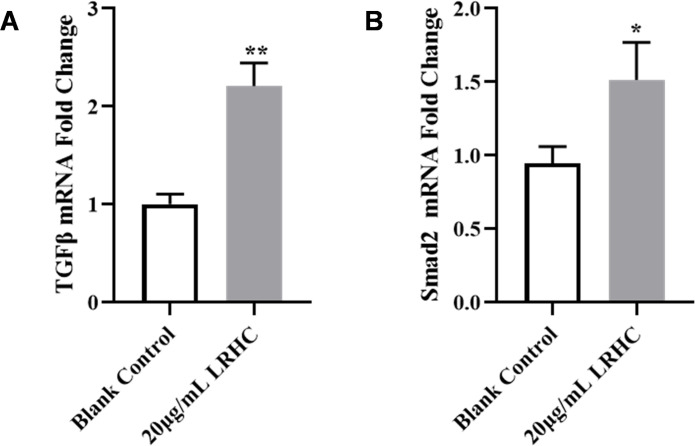
The effects of LRHC on TGF-β signaling pathway in FBs. The levels of **(A)** TGF-β mRNA, **(B)** Smad2 mRNA are measured by RT-qPCR assay. Statistical analyses were performed using a two-way ANOVA with Tukey multiple comparisons test. Data are presented as mean ± SEM (n = 3). *p < 0.05, **p < 0.01 *vs.* the Blank Control.

Previous studies report that TGF-β pathway activation promotes extracellular matrix (ECM) production. To validate LRHC’s regulatory role, qRT-PCR analysis confirmed significantly elevated mRNA levels of TGF-β (Fig 8A) and *Smad2* (Fig 8B) in LRHC-treated fibroblasts *vs.* blank controls (*p* < 0.05), consistent with transcriptomic profiling data. Thus, LRHC enhances ECM synthesis through TGF-β signaling and stabilizes cytoskeletal architecture *via* adherens junction pathways, collectively mediating anti-aging effects.

## Discussion

Collagen and hydrolyzed collagen of animal origin are recognized as effective nutraceuticals for skin anti-aging [14]. However, the application of animal-derived collagen and collagen peptides in skin topical preparations is limited due to their high molecular weight or the uncertainty of immunogenicity and virus [31,32]. Genetic engineering technology offers a promising approach to synthesize low-molecular-weight human-derived collagen, enabling the development of collagen-based topical preparations, as only small collagen fragments can achieve effective transdermal delivery. In this study, we developed the low molecular weight LRHC using synthetic biology combined with a specific enzymatic hydrolysis technique. The LRHC was designed by repeating a stable, highly active fragment containing suitable enzymatic cleavage sites, based on the template of human type III collagen, followed by precise enzymatic processing to obtain the final product. However, research on the skin permeability and anti-aging efficacy of topically administered collagen remains limited, warranting further investigation.

In proliferation, migration, and adhesion assays, LRHC demonstrated fundamental biological activity comparable to or even superior to full-length bovine type I collagen of animal origin. Both *in vitro* and murine studies confirmed the efficacy of LRHC in combating skin aging, showing that it upregulated the expression of COL1, COL3, and ELN in human dermal fibroblasts and enhanced basement membrane protein expression in photoaged mice, ultimately improving collagen fiber density. Notably, our study demonstrated that LRHC could penetrate into the dermis within 4 hours of topical application, suggesting its ability to traverse the human stratum corneum and reach deeper skin layers. Mechanistically, RNA-seq analysis indicated that LRHC exerts its anti-aging effects by promoting extracellular matrix (ECM) production *via* the TGF-β signaling pathway and maintaining cytoskeletal integrity through the adherens junction pathway. In summary, LRHC can also be applied in topical formulations and is a promising component for resisting skin aging.

Currently, obtaining full-length human-derived collagen remains challenging, so we chose bovine collagen with 97.47% homology to human collagen for basic biological activity comparisons. The development of synthetic biology is expected to enable us to obtain full-length human-derived collagen, which can be used for further research. In addition, it is noteworthy that the LRHC examined in our study represents a type III collagen peptide, and there can be significant variations among different types of collagen [33]. For permeability evaluation, we utilized Bama miniature pig skin – although not perfectly replicating human skin, this model is well-established as a valid alternative for permeability studies [34,35]. However, the high protein content and complex composition of porcine skin presented analytical challenges, as neither HPLC nor UV-VIS spectroscopy could completely eliminate interference from endogenous proteins, thereby precluding precise determination of residual LRHC content. Regarding collagen, the upper limit of molecular weight that can penetrate the skin barrier and the differences in anti-aging effects between different molecular fragments are still under investigation. Resolution of these issues will significantly expand collagen’s applications in topical formulations, particularly in the cosmetic industry, where its potential is especially promising.

## Supporting information

S1 Raw images Uncropped raw gel images. (PDF)
