# Supplementary material for: Anti-aging effect of low molecular weight recombinant humanized collagen on photo-aging by activating adherence junction signaling pathways
Source: PLoS One. 2025 Aug 29;20(8):e0329460. doi: 10.1371/journal.pone.0329460 (PMC12396721; doi:10.1371/journal.pone.0329460)
Supplement: S1 Raw images — (PDF) [file pone.0329460.s001.pdf]

Figure 1
Main figure

A

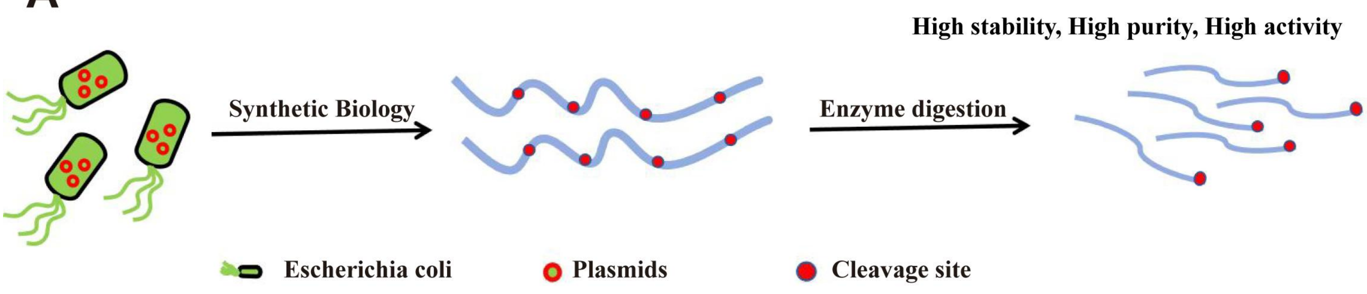

B

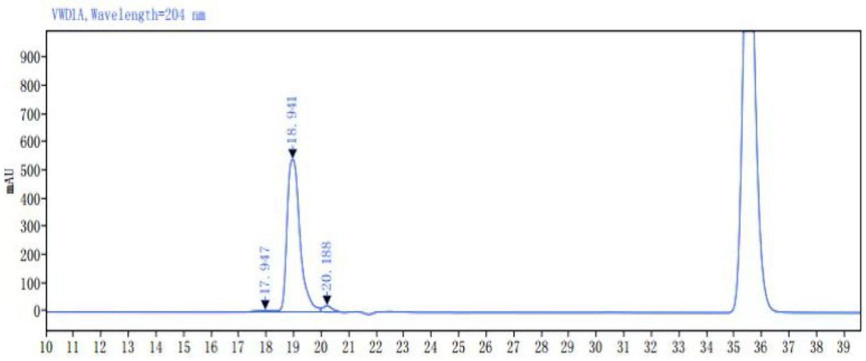

C

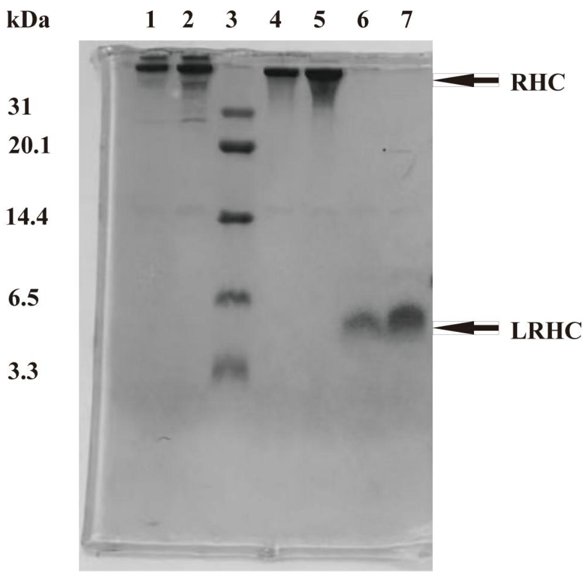

Lane 1: Standard bovine serum albumin (BSA)
(Protein load: 2 μg)
Lane 2: Standard bovine serum albumin (BSA)
(Protein load: 4 μg)
Lane 3: Protein-molecular-weight size markers
Lane 4: Type III recombinant human collagen (RHC)
(Protein load: 2 μg)
Lane 5: Type III recombinant human collagen (RHC)
(Protein load: 4 μg)
Lane 6: Low molecular weight recombinant humanized collagen (LRHC)
(Protein load: 2 μg)
Lane 7: Low molecular weight recombinant humanized collagen (LRHC)
(Protein load: 4 μg)

Figure 1
Supplemental figures-raw gel image

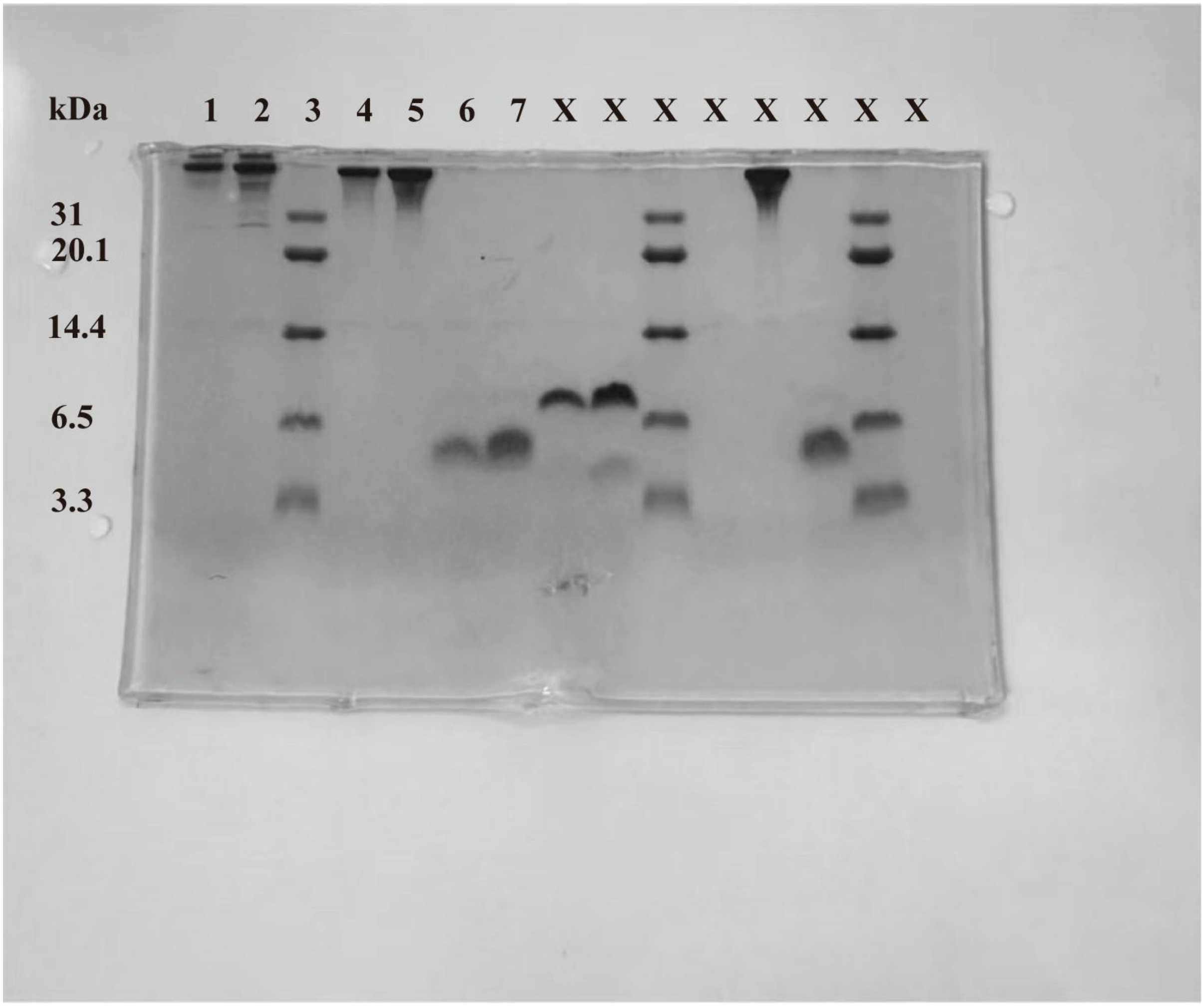

Lane 1: Standard bovine serum albumin (BSA)
(Protein load: 2 μg)
Lane 2: Standard bovine serum albumin (BSA)
(Protein load: 4 μg)
Lane 3: Protein-molecular-weight size markers
Lane 4: Type III recombinant human collagen (RHC)
(Protein load: 2 μg)
Lane 5: Type III recombinant human collagen (RHC)
(Protein load: 4 μg)
Lane 6: Low molecular weight recombinant humanized collagen (LRHC)
(Protein load: 2 μg)
Lane 7: Low molecular weight recombinant humanized collagen (LRHC)
(Protein load: 4 μg)

Figure 1
Supplemental figures-Raw gel image-Related parts of the article

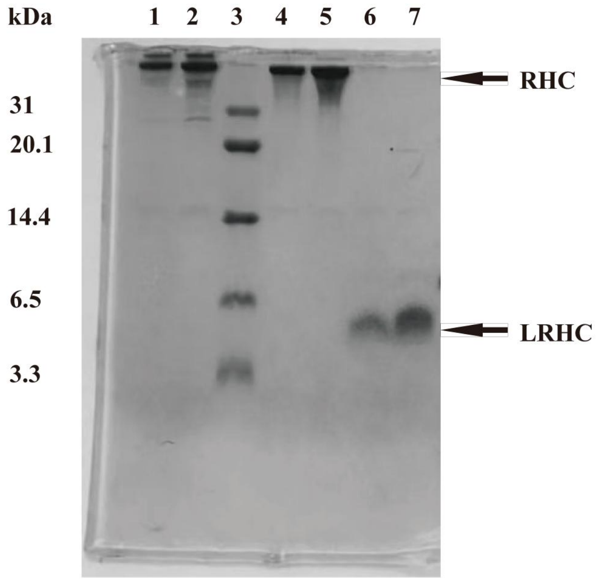

Lane 1: Standard bovine serum albumin (BSA)
(Protein load: 2 μg)
Lane 2: Standard bovine serum albumin (BSA)
(Protein load: 4 μg)
Lane 3: Protein-molecular-weight size markers
Lane 4: Type III recombinant human collagen (RHC)
(Protein load: 2 μg)
Lane 5: Type III recombinant human collagen (RHC)
(Protein load: 4 μg)
Lane 6: Low molecular weight recombinant humanized collagen (LRHC)
(Protein load: 2 μg)
Lane 7: Low molecular weight recombinant humanized collagen (LRHC)
(Protein load: 4 μg)
